# 

**Published:** 1896-11-21

**Authors:** 


### EARLY EDITION.

**Notices of Births, Deaths, and Marriages** are inserted in this column at a charge of 2s. 6d. for an announcement not exceeding four lines.

### NOTICE TO CORRESPONDENTS.

All MS., Letters, Books for Review, and other matters intended for the Editor should be addressed THE EDITOR, "THE HOSPITAL," THE HOSPITAL BUILDING, 28 & 29, SOUTHAMPTON STREET, STRAND, W.C.

The Editor cannot undertake to return rejected MS., even when accompanied by stamped directed envelope.

**Notice to Advertisers and Subscribers.**

All Advertisements, Orders for Copies of THE HOSPITAL, and Business Communications should be addressed to **THE MANAGER, (Not to The Editor),** THE HOSPITAL, The Hospital Building, 28 & 29, Southampton Street, Strand, London, W.C.

The Cost of Subscription, post free, is as follows:—Per week, 2½d.; per quarter, 2s. 8d.; per half-year, 5s. 3d.; per annum, 10s. 6d. Foreign:—Per annum, 15s. 2d.; payable, in all cases, in advance.

**IMPORTANT NOTICE.**

The EDITORIAL and PUBLISHING OFFICES of "THE HOSPITAL" are now TRANSFERRED to their new premises, THE HOSPITAL BUILDING, 28 & 29, SOUTHAMPTON STREET, STRAND, LONDON, W.C.

**NOTICE.—Vol. XX. of "The Hospital" (April to September, 1896), in handsome cloth binding, is now on sale at the Office of this Journal, price 7s. 6d. Cases for binding the Numbers contained in this Volume 1s. 6d. Index to Vol. XX., 2½d., post free. The Monthly Part for November is now ready, Price 6d., by post 8d.**

### Scraps and Gleanings.

The result of the past year's work at the Plymouth Public Dispensary is an overdrawn balance at the bank of £179. Three thousand seven hundred and seventy-eight patients were treated against 3,615 in the previous year. The income amounted to £937, and the expenditure to £1,039.

Fifty-six patients (against 30 in the previous year) were treated at the Fleetwood Cottage Hospital in the year 1895-6. Receipts for the year amounted to £302, and expenses to £285.

### BOOKS RECEIVED.

J. and A. Churchill.

"The Practice of Midwifery." By D. Lloyd Roberts, M.D., F.R.S., Edin., F.R.C.P.

Macmillan and Co.

"Elementary Practical Physiology and Histology." By Foster and Langle.

John Wright and Co.

"Lectures in Renal and Urinary Diseases." By Robert Saundby, M.D.

**Periodicals.**—The Quiver, The Medical Bulletin, The Scalpel, The Sunday at Home, The Leisure Hour, The New York Medical Journal.

### THE STUDENT OF MEDICINE.

### APPOINTMENTS.

G. L. Atkinson, L.S.A.Lond., appointed House Surgeon to King's College Hospital. F. S. Barnett, M.R.C S., L.S.A.Lond., L F.P.S.Glas, appointed Medical Officer and Public Vaccinator for the Lewisham District of the Lewisham Union. L. B. Betts, M.R.C.S., L.R.C.P.Lond., appointed House Surgeon to the Westminster Hospital. J. S. Boyd, L.R.C.P., L.R.C.S. Edin., appointed Medical Officer for the Sixth District of the West Ham Union. J. L. Burch, M.D., M.R.C.P., appointed Assistant Physician to the North London Hospital for Consumption. H. A. Crutwell, L.R.C.P., L.R.C.S.Edin., appointed Medical Officer for the Sixth District of the Market Harborough Union. A. R. Darley, M.D., appointed Medical Officer for the Sixth District of the Daventry Union. H. Dingle, M.R.C.S., L.R.C.P., appointed House Surgeon to King's College Hospital. C. C. J. Erhardt, M.R.C.S., L.R.C.P., appointed Assistant House Accoucheur to King's College Hospital. G. W. R. Fernando, M.B., C.M., appointed House Surgeon to the Birmingham and Midland Ear and Throat Hospital. W. J. Forbes, M.B., B Ch., B.A.O., R.U.I., appointed Medical Officer to the Scriven District of the Knaresborough Union. F. R. Greenwood, M.R.C.S.Eng., L.R.C.P.Lond., appointed Resident Obstetric and Ophthalmic House Surgeon at the Queen's Hospital, Birmingham. J. C. Griffith, B.Sc., M.R.C.S.Eng., L.R C.P. Lond, appointed House Physician at the Queen's Hospital, Birmingham. E B. H. Hughes, L R.C.P.E, L.R.C.S.E, and L F P S Glas., appointed Assistant Medical Officer to the Newcastle-on-Tyne Dispensary. F. H. Jacob, M.R C.S., L R.C.P, appointed House Physician to King's College Hospital. A E Jerman, M.R C S, L R C.P.Lond, appointed Assistant House Surgeon to the Westminster Hospital. J. Kerr, M.A., M.D., D.P.H.Cantab., appointed Honorary Medical Officer to the Bradford Infirmary. J. A. Lorimer, L.R.C.P.Lond., M.R.C.S.Eng, appointed Medical Officer to the Farnham and Hartley Wintney District School. T. M. Martin, M.B., L R.C P., L.R.C.S.Edin., L.F.P.S.Glasg., appointed Medical Officer and Public Vaccinator for the Parish of Lochwinnoch. A. McDougall, M.B. (Vic.), M.R.C.S, appointed Senior House Surgeon at the Ancoats Hospital. A. MacGregor, M.D., appointed Assistant Physician to the North London Hospital for Consumption and Diseases of the Chest. E. Playfair, M.B.Lond., M.R.C S, L.R.C.P., appointed Medical Registrar to King's College Hospital. C. Pollard, appointed Medical Officer for the Holt District of the Martley Union. J. H. Powell, L.R.C.S., L.R.C.P.Edin., appointed Second Assistant Medical Officer to St. George's Infirmary, Fulham Road, S.W. E. S. Robinson, L.R C.P., M.R.C.S., appointed Medical Officer for the Astley District of the Martley Union. E. A. Smith, M.B, Ch.B.Vict., M.R.C.S.Eng., L.R.C.P.Lond., appointed Medical Tutor and Registrar Liverpool Royal Infirmary. G. U. Smith, M.B.Lond., M.R.C.S., L.R.C.P., appointed Assistant House Physician to King's College Hospital. E. C. Thomas, M.B., C.M.Edin., appointed Medical Officer of Health for the Lampeter Rural District. P. C. E. Tribe, M.R.C.S., L.R C.P., appointed House Accoucheur to King's College Hospital. G. F. Welsford, B.A.Camb, M.B, M.R C S.Eng, appointed Medical Officer for the Washfield District of the Tiverton Union. Catherine M. Wickham, appointed Zenana Medical Officer at Rajkot, Kathiawar, India. R. P. Williams, M.R.C.S, L R C.P., appointed House Surgeon to King's College Hospital. W. S. Willmore, M.R C.S Eng, L R C.P.Lond., appointed House Surgeon at theQueen's Hospital, Birmingham.

